# The Serine Phosphatase SerB of *Porphyromonas gingivalis* Suppresses IL-8 Production by Dephosphorylation of NF-κB RelA/p65

**DOI:** 10.1371/journal.ppat.1003326

**Published:** 2013-04-18

**Authors:** Hiroki Takeuchi, Takanori Hirano, Sarah E. Whitmore, Ichijiro Morisaki, Atsuo Amano, Richard J. Lamont

**Affiliations:** 1 Center for Oral Health and Systemic Disease, School of Dentistry, University of Louisville, Louisville, Kentucky, United States of America; 2 Division of Special Care Dentistry, Osaka University Dental Hospital, Suita-Osaka, Japan; 3 Department of Preventive Dentistry, Osaka University Graduate School of Dentistry, Suita-Osaka, Japan; Tufts University School of Medicine, United States of America

## Abstract

*Porphyromonas gingivalis* is a major pathogen in severe and chronic manifestations of periodontal disease, which is one of the most common infections of humans. A central feature of *P. gingivalis* pathogenicity is dysregulation of innate immunity at the gingival epithelial interface, including suppression of IL-8 production by epithelial cells. NF-κB is a transcriptional regulator that controls important aspects of innate immune responses, and NF-κB RelA/p65 homodimers regulate transcription of IL8. Phosphorylation of the NF-κB p65 subunit protein on the serine 536 residue affects nuclear translocation and transcription of target genes. Here we show that SerB, a haloacid dehalogenase (HAD) family serine phosphatase secreted by *P. gingivalis*, is produced intracellularly and can specifically dephosphorylate S536 of p65 in gingival epithelial cells. A *P. gingivalis* mutant lacking SerB was impaired in dephosphorylation of p65 S536, and ectopically expressed SerB bound to p65 and co-localized with p65 in the cytoplasm. Ectopic expression of SerB also resulted in dephosphorylation of p65 with reduced nuclear translocation in TNF-α-stimulated epithelial cells. In contrast, the p105/50 subunit of NF-κB was unaffected by SerB. Co-expression of a constitutively active p65 mutant (S536D) relieved inhibition of nuclear translocation. Both the activity of the IL8 promoter and production of IL-8 were diminished by SerB. Deletion and truncation mutants of SerB lacking the HAD-family enzyme motifs of SerB were unable to dephosphorylate p65, inhibit nuclear translocation or abrogate IL8 transcription. Specific dephosphorylation of NF-κB p65 S536 by SerB, and consequent inhibition of nuclear translocation, provides the molecular basis for a bacterial strategy to manipulate host inflammatory pathways and repress innate immunity at mucosal surfaces.

## Author Summary

Periodontal diseases are one of the most common infections of humans, and are characterized by gingival inflammation and destruction of the hard and soft tissues that support the tooth, eventually causing tooth loss. *Porphyromonas gingivalis* is a major pathogen in periodontal diseases and a key pathogenic attribute of this organism is the ability to disrupt host innate immunity. Infection of gingival epithelial cells by *P. gingivalis* suppresses production of the neutrophil chemokine IL-8. This inhibitory process is associated with the *P. gingivalis* serine phosphatase, SerB. In this study we show that SerB has a potent and specific ability to inhibit activation the NF-κB transcription factor which regulates IL-8 production. Mechanistically, SerB binds to and dephosphorylates the p65 subunit of NF-κB which prevents nuclear translocation and subsequent transcription of the IL8 gene. Targeting the NF-κB p65 subunit allows *P. gingivalis* to dampen IL-8 dependent inflammatory responses, facilitate survival and potentially to establish a favorable niche for the entire periodontal microbial community.

## Introduction

Many of the mucosal surfaces of humans are colonized by a diverse and abundant microbiota. In most instances the host remains healthy, in large part due to numerous innate and acquired immune mechanisms that limit microbial intrusion and rapidly kill organisms that traverse epithelial barriers. In the periodontal tissues of the oral cavity the epithelium of the subgingival compartment plays a central role in orchestration of innate immunity. While this tissue is relatively porous, gingival epithelial cells secrete high levels of IL-8 and consequently large numbers of neutrophils are recruited into the periodontal area where they serve to constrain the microbial challenge [1]. Successful periodontal pathogens, such as *Porphyromonas gingivalis*, are capable of disrupting innate defenses, and indeed one virulence determinant of *P. gingivalis* is inhibition of IL-8 production by gingival epithelial cells, a strategy known as localized chemokine paralysis [2], [3]. Moreover, periodontal diseases are multispecies infections involving pathogenic communities in which the microbial constituents exhibit polymicrobial synergy. Consistent with this, *P. gingivalis* can antagonize IL-8 secretion in the presence of stimulatory organisms [2], a property that will allow *P. gingivalis* to enhance the pathogenicity of the entire multispecies periodontal community and which contributes to its designation as a keystone pathogen [4]. The *P. gingivalis* serine phosphatase SerB is required for IL-8 suppression, and in a murine model of disease a mutant lacking SerB induces higher levels of neutrophil recruitment into gingival tissues compared to the parental strain [5]. Additionally, loss of SerB attenuates alveolar bone destruction in animal infection models demonstrating that SerB, and its associated anti-inflammatory action, is required for *P. gingivalis* to realize its full pathogenic potential [5]. The mechanistic basis for the SerB-dependent inhibition of IL-8 remains undetermined.

*P. gingivalis* is an intracellular pathogen and epithelial cell entry is accomplished by a very limited number of bacterial effectors. The major fimbriae mediate attachment to integrin receptors and this leads to remodeling of the host cell cytoskeleton. SerB is secreted by *P. gingivalis* and facilitates invasion through dephosphorylation and activation of the host actin depolymerizing protein cofilin [6], [7], [8]. SerB is a haloacid dehalogenase (HAD) family enzyme that is functionally versatile and can impact the dynamics of both the host microfilament and the microtubule cytoskeleton [8], [9]; however, the complete range of host cell serine phosphoproteins that can be dephosphorylated by the enzyme is unknown.

At the transcriptional level, the predominant control over IL8 is exerted by the eukaryotic transcription factor NF-κB [10]. NF-κB can be comprised of homo- or hetero-dimers containing combinations of the subunits RelA/p65, c-Rel, RelB, p100, p105 and p50, each of which contains a Rel homology domain that mediates subunit binding [10]. The non-Rel NF-κB subunit, RPS3, guides NF-κB to specific κB sites on the chromosome and contributes to regulatory specificity [11]. In unstimulated cells, NF-κB is generally sequestered in the cytosol and bound to inhibitory IκB proteins [12]. In the canonical pathway stimuli, including bacterial products and proinflammatory cytokines, activate IκB kinases (IKKα and β, along with the regulatory subunit IKKγ) that phosphorylate IκB and induce ubiquitination and proteosomal degradation of IκB [13], [14]. The free homo- or hetero-dimer complex then rapidly enters the nucleus and initiates transcription of downstream effector genes [15]. Noncanonical activation pathways also exist in which there is no sequestration of NF-κB by IκB proteins, and in one example active p52-RelB complexes can be generated by IKKα-mediated phosphorylation of p100 [16].

Many considerations contribute to the transcriptional activation of NF-κB target genes, including the nature of the homo- or hetero-dimers, the involvement of basal transcription factors and coactivators, and histone modifications in the promoter region [10]. In addition, posttranslational modifications of NF-κB subunits, including phosphorylation, acetylation and methylation, have been shown to contribute to regulation of transcriptional activity [17]. The p65 subunit, for example, can be phosphorylated on serine (S) 276 in the Rel homology domain as well as on S468 and S536 in the C-terminal transcriptional activation domain, in response to a variety of stimuli [17]. Phosphorylation on these sites can have different functional consequences depending on the nature of the stimulus, the kinase involved and the target gene. S536 can be phosphorylated by IKKs in response to TNF-α, LPS, *Helicobacter pylori* or HTLV1 TAX protein, resulting in elevated function [17]. The enhanced transcriptional activity following p65 S536 phosphorylation may ensue from a conformational change affecting binding to other subunits or decreased affinity for the IκBα inhibitor [18], [19]. S536 phosphorylation can also impact the kinetics of nuclear import of NF-κB p65 and cytoplasmic retention of IκBα [20]. Furthermore, there is evidence that S536 phosphorylation represents a noncanonical activation pathway whereby phosphorylated p65 can translocate to the nucleus independent of IκBα regulation [21].

In this study we found that SerB-mediated inhibition of IL-8 production by epithelial cells involved suppression of NF-κB activation. SerB was capable of binding to the p65 subunit of NF-κB and dephosphorylated p65 at S536 through the action of the HAD-family enzyme motifs. SerB thus prevented nuclear translocation of NF-κB p65 and subsequent transcription of the IL8 gene. This work provides insights into both the immune-subversive processes of *P. gingivalis*, and the development of polymicrobial synergy in pathogenic communities.

## Results

### *P. gingivalis* dephosphorylates NF-κB p65 in a SerB dependent manner

**Figure 1:**
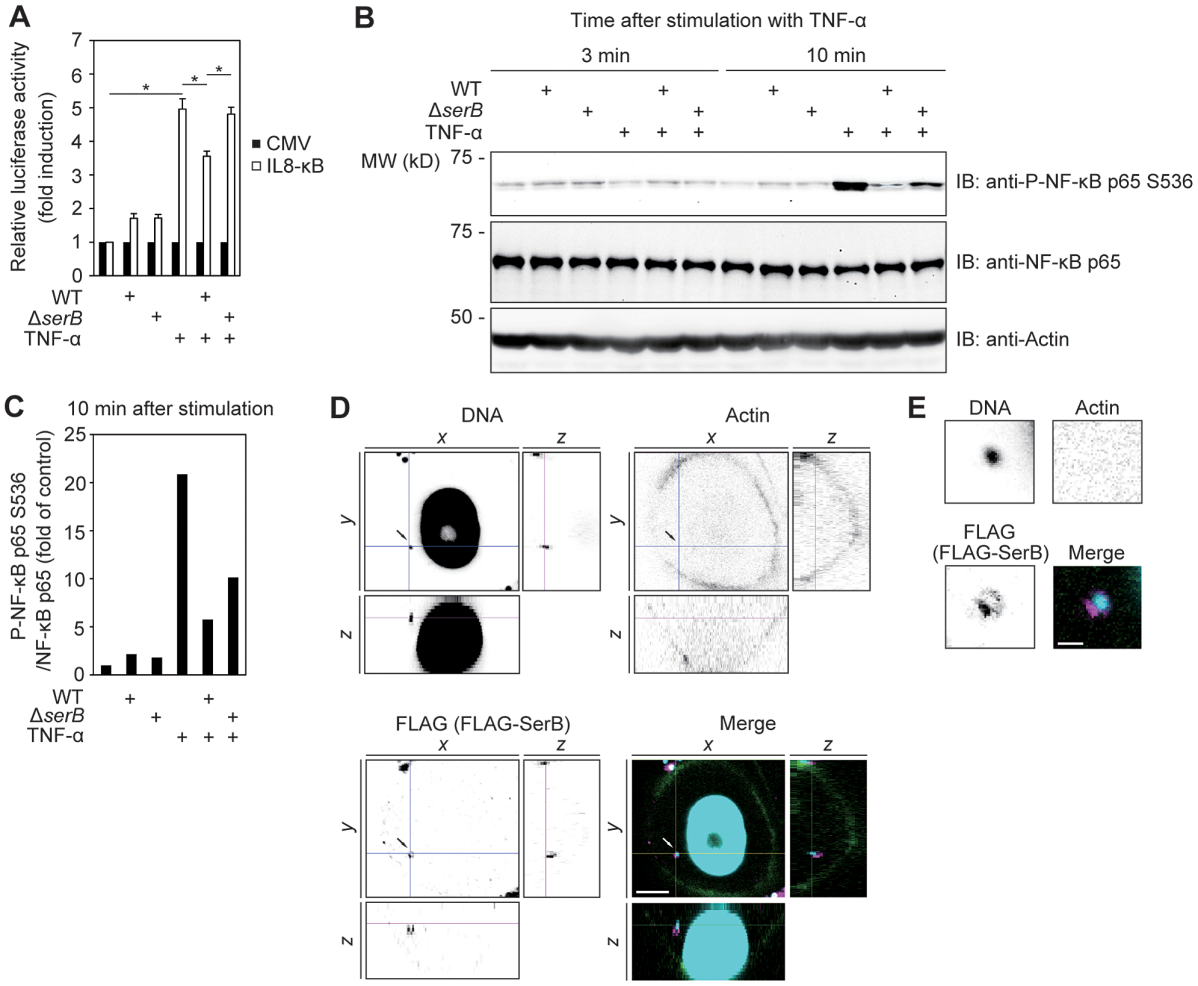
Infection of TIGK epithelial cells with *P. gingivalis* expressing SerB diminishes IL8 promoter activity and phosphorylation of NF-κB p65. (**A**) TIGKs were transiently transfected with the pIL-8 κB-Luc luciferase reporter plasmid for IL8 promoter activity, or pRL-CMV (CMV) in which Renilla luciferase is driven by the cytomegalovirus promoter, and infected with *P. gingivalis* WT or Δ*serB* mutant at a MOI of 10. At 16 h after infection, TIGKs were stimulated with TNF-α (10 ng/ml) for 3 h or left unstimulated and luciferase activity was measured and normalized to Renilla luciferase. Results are presented as fold relative to the activity of the non-stimulated control. Values are mean ± SD; n = 3; *, p<0.05. Data shown are representative of 2 biological replicates. (**B**) Immunoblots (IB) of lysates of TIGKs infected with *P. gingivalis* WT or Δs*erB* at a MOI of 10. At 16 h after infection, TIGKs were stimulated with TNF-α (10 ng/ml) or left unstimulated for the indicated time periods prior to cell lysis. Blots were probed with the antibodies indicated. Actin was used as a loading control. Result is representative of 2 biological replicates. (**C**) Densitometry of immunoblot in B) showing ratio of phospho-NF-κB p65 (S536) relative to total immunodetectable NF-κB p65. (**D**) Confocal microscopy showing expression of SerB by intracellular *P. gingivalis*. TIGKs were infected with *P. gingivalis* expressing FLAG-SerB at a MOI of 10 for 2 h, and stained with DAPI (cyan), fluorescein isothiocyanate (FITC)-phalloidin (green) or anti-FLAG (magenta). Bar = 5 µm. (**E**) Higher magnification of *P. gingivalis* indicated by arrow in **D**). Bar = 1 µm.

Previous reports have established that *P. gingivalis* can inhibit the accumulation of IL-8 in the supernatants of gingival epithelial cells [2]. The secreted serine phosphatase SerB is required for maximal inhibition of IL-8 production [9], and to explore whether regulation of IL-8 by SerB occurs at the transcriptional level we transfected telomerase immortalized gingival keratinocytes (TIGKs) with the reporter plasmid pIL-8κB-Luc in which the luciferase gene is controlled by the NF-κB-regulated IL8 promoter. Transfected cells were then infected with *P. gingivalis* ATCC 33277 wild type (WT) or its isogenic Δ*serB* mutant at MOI 10 for 16 h, stimulated with tumor necrosis factor (TNF-α) and assayed for luciferase activity at 3 h after stimulation (Figure 1A). IL8 promoter activity was increased 5-fold by TNF-α stimulation, and infection with *P. gingivalis* WT significantly reduced IL8 promoter activity to 3.6-fold over unstimulated basal levels. In contrast, the Δ*serB* mutant was unable to antagonize TNF-α stimulated IL8 promoter activity. These results suggest that the ability of SerB to diminish IL-8 secretion by epithelial cells involves inhibition of NF-κB dependent transcription of the IL8 gene.

Recent reports have documented that phosphorylation of NF-κB p65 at the S536 residue in Jurkat cells induces IL8 transcription following stimulation by ionomycin [21]. We hypothesized therefore that p65 S536 might be a target for dephosphorylation by *P. gingivalis* SerB. Immunoblots (Figure 1B) with scanning densitometry (Figure 1C) showed that after 10 min of TNF-α stimulation the levels of p65 S536 phosphorylation in TIGKs were reduced over 4-fold by *P. gingivalis* WT. The Δ*serB* mutant had a diminished capacity to reduce the phosphorylation of the NF-κB p65 S536 residue. *P. gingivalis* expressing the SerB enzyme can thus induce dephosphorylation of p65 S536.

*P. gingivalis* is efficiently internalized by gingival epithelial cells [22], [23], and intracellular invasion is required for NF-κB suppression [2]. To establish that SerB is produced and secreted by intracellular *P. gingivalis*, we infected TIGKs with *P. gingivalis* expressing SerB fused to a FLAG epitope [6]. Intracellular bacteria were detected with DAPI, to avoid antibody reaction with extracellular antigens. The detection of infected bacteria with DAPI was found to be as efficient and specific as with *P. gingivalis* antibodies (Figure S1). As shown in Figures 1D and 1E, confocal microscopy with FLAG antibodies detected SerB within the cytoplasm of TIGKs. Additionally, fractionation of *P. gingivalis* infected TIGK cells showed that SerB could be detected in both the membrane and cytosol compartments (Figure S2). Hence, SerB is accessible for direct interactions with host cytosolic phosphoproteins following *P. gingivalis* infection.

### SerB binds to and dephosphorylates NF-κB p65

**Figure 2:**
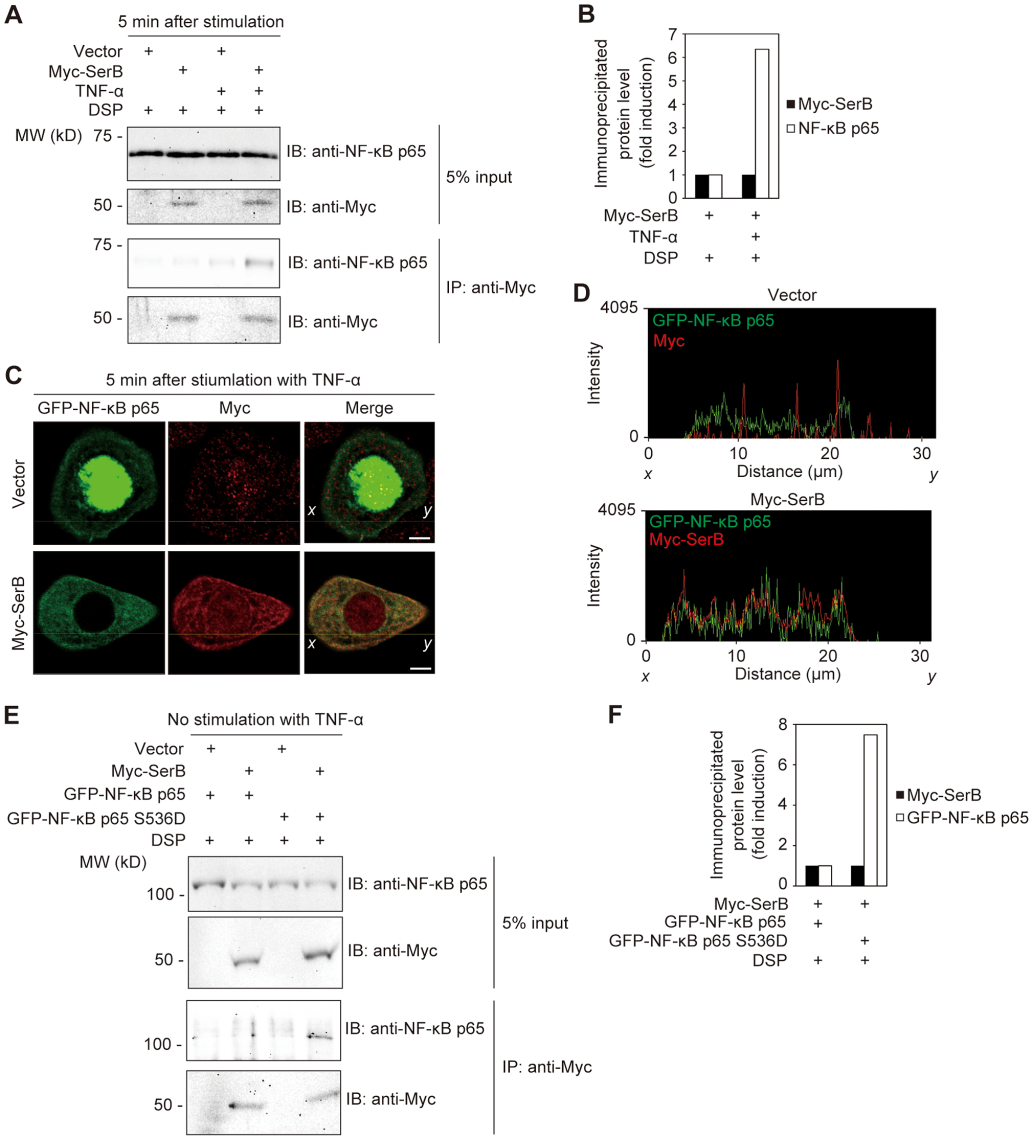
Ectopically expressed SerB binds to and dephosphorylates NF-κB p65. (**A**) TIGKs transfected with empty vector or Myc-SerB were stimulated with TNF-α (10 ng/ml) for 5 min or left unstimulated. Cells were cross-linked with DSP and cell lysates were immunoprecipitated with anti-Myc antibody. Immunoblots (IB) show cell lysates prior to immunoprecipitation (5% input) and immunoprecipitate (IP) with Myc antibodies. Result is representative of 3 biological replicates. (**B**) Densitometry of immunoblot in A) showing ratio of immunoprecipitated NF-κB p65 relative to immunoprecipitated Myc-SerB. (**C**) Confocal microscopic images of TIGKs expressing GFP-NF-κB p65 (green) and either Myc (Vector) or Myc-SerB. At 36 h after transfection, cells were stimulated with TNF-α (10 ng/ml) for 5 min or left unstimulated. Cells were fixed and stained with anti-Myc (red). Bars = 5 µm. Data shown are representative of 2 biological replicates. (**D**) The intensity (Olympus FluoView software) of the fluorescence signals of GFP-NF-κB p65 (green) and either of Myc (red, Vector) or Myc-SerB (red) on the *x*-*y* lines indicated in C) are shown. (**E**) TIGKs transfected with empty vector or Myc-SerB, and GFP-NF-κB p65 or GFP-NF-κB p65 S536D, were cross-linked with DSP and cell lysates were immunoprecipitated with anti-Myc antibody. Immunoblots (IB) show cell lysates prior to immunoprecipitation (5% input) and immunoprecipitate (IP) with Myc antibodies. Result is representative of 2 biological replicates. (**F**) Densitometry of immunoblot in E) showing ratio of immunoprecipitated GFP-NF-κB p65 or p65 S536D relative to immunoprecipitated Myc-SerB.

To investigate the function of SerB independent of other *P. gingivalis* molecules, we constructed a mammalian expression vector bearing a fusion protein, Myc-SerB, which was transiently expressed in TIGK cells. Using the cross-linking agent DSP to stabilize enzyme-substrate interactions, we found that Myc-SerB, but not Myc alone (empty vector), co-immunoprecipitated with endogenous NF-κB p65 (Figures 2A and 2B). Co-precipitation, albeit less efficient, was also observed in the absence of cross-linking reagent (Figure S3). These results indicate that SerB can interact directly with p65 or a p65 complex, but do not exclude the possibility that other *P. gingivalis* molecules can also interact with NF-κB components.

To further examine the interaction of SerB with NF-κB p65, GFP tagged NF-κB p65 was generated and introduced into TIGK cells along with Myc-SerB. We then visualized co-localization of Myc-SerB and GFP-NF-κB p65 in TIGKs. Figures 2C and 2D show that when Myc-SerB and GFP-NF-κB p65 were ectopically expressed in TIGKs, Myc-SerB co-localized with GFP-NF-κB p65 in the cytoplasmic area. We then confirmed that the phosphorylation status of NF-κB p65 is necessary for SerB binding by constructing a constitutively-active phosphomimic p65 S536D [21]. TIGKs were transfected with GFP-NF-κB p65 or GFP-NF-κB p65 S536D, along with Myc-SerB, and left unstimulated to maintain the phosphorylation of wild type p65 at basal levels. Immunoprecipitation with Myc antibodies revealed that Myc-SerB co-immunoprecipitated with GFP-NF-κB p65 S536D whereas co-precipitation of Myc-SerB with wild type, unphosphorylated NF-κB p65 was below detection thresholds (Figures 2E and 2F). This finding demonstrates that the phosphorylation status of NF-κB p65 S536 is significant for the binding of SerB to NF-κB p65.

**Figure 3:**
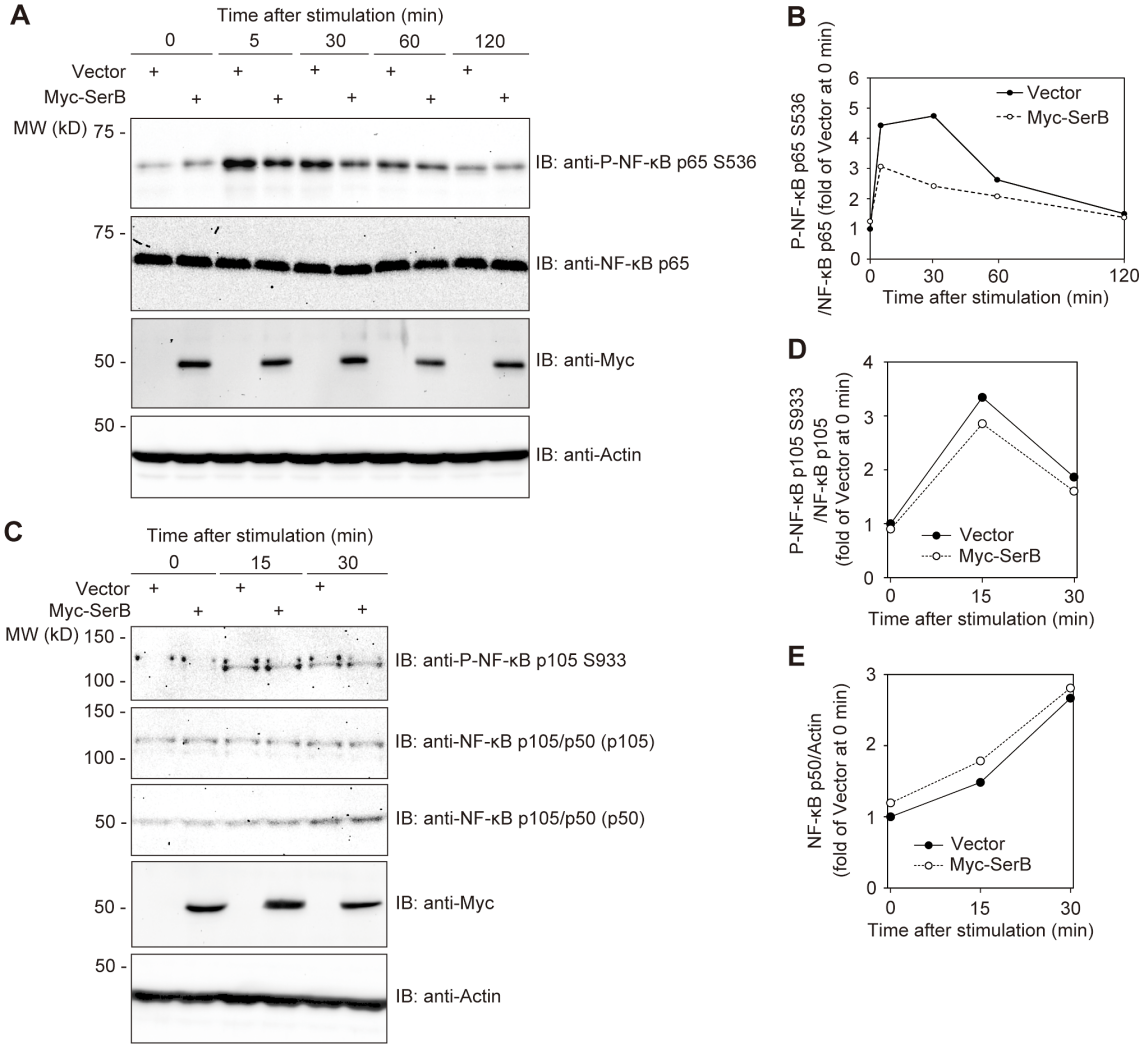
SerB dephosphorylates NF-κB p65 but not p105. (**A**) TIGKs were transfected with empty vector or Myc-SerB. At 36 h after transfection, cells were stimulated with TNF-α (5 ng/ml), and at the time periods shown cell extracts were prepared and immunoblotted (IB) with the antibodies indicated. Actin was used as a loading control. Result is representative of 3 biological replicates. (**B**) Densitometry of immunoblot in A) showing ratio of phospho-NF-κB p65 (S536) relative to total immunodetectable NF-κB p65. (**C**) TIGKs were transiently transfected with empty vector or Myc-SerB. At 36 h after transfection, cells were stimulated with TNF-α (5 ng/ml), and at the time periods shown cell extracts were prepared immunoblotted (IB) with the antibodies indicated. Result is representative of 3 biological replicates. (**D**) Densitometry of immunoblot in C) showing ratio of phospho-NF-κB p105 (S933) relative to total immunodetectable NF-κB p105. (**E**) Densitometry of immunoblot in C) showing ratio of phospho-NF-κB p50 relative to actin.

Next we investigated whether binding of SerB to p65 effectuates dephosphorylation of S536. Myc-SerB was ectopically expressed in TIGKs and after stimulation with TNF-α, the kinetics of NF-κB p65 phosphorylation were examined by immunoblot analysis with quantitative densitometry. As shown in Figures 3A and 3B, TIGKs expressing Myc-SerB displayed a lower level of phosphorylated NF-κB p65 from 5–30 min after TNF-α stimulation compared to cells transfected with empty vector. After 1 h, levels of phospho-p65 declined in cells expressing both Myc-SerB and Myc alone as a result of the transient effect of TNF-α. These findings indicate that SerB can dephosphorylate p65 at the S536 residue. To address the specificity of SerB action, we investigated whether the enzyme can also dephosphorylate the S276 and S468 residues. SerB did not significantly decrease the level of phospho-NF-κB p65 S276 or S468 induced by TNF-α stimulation (Figures S4 and S5). In addition, the effect of SerB on phosphorylation of the NF-κB p105 subunit at S933 was assessed. Similarly, SerB did not dephosphorylate the NF-κB p105 S933 residue (Figures 3C and 3D). Phosphorylation of NF-κB p105 leads to processing into the p50 subunit [24], [25] and hence we also confirmed that the amount of NF-κB p50 was unchanged in TNF-α stimulated TIGKs expressing Myc-SerB compared to the empty vector control (Figures 3C and 3E). Thus, SerB does not impact the phosphorylation status of NF-κB p65 S276, p65 S468, or p105 S933, and does not affect processing of phospho-NF-κB p105 into NF-κB p50. The action of SerB on NF-κB thus appears to be specific for p65 S536.

### SerB prevents the nuclear translocation of NF-κB p65

**Figure 4:**
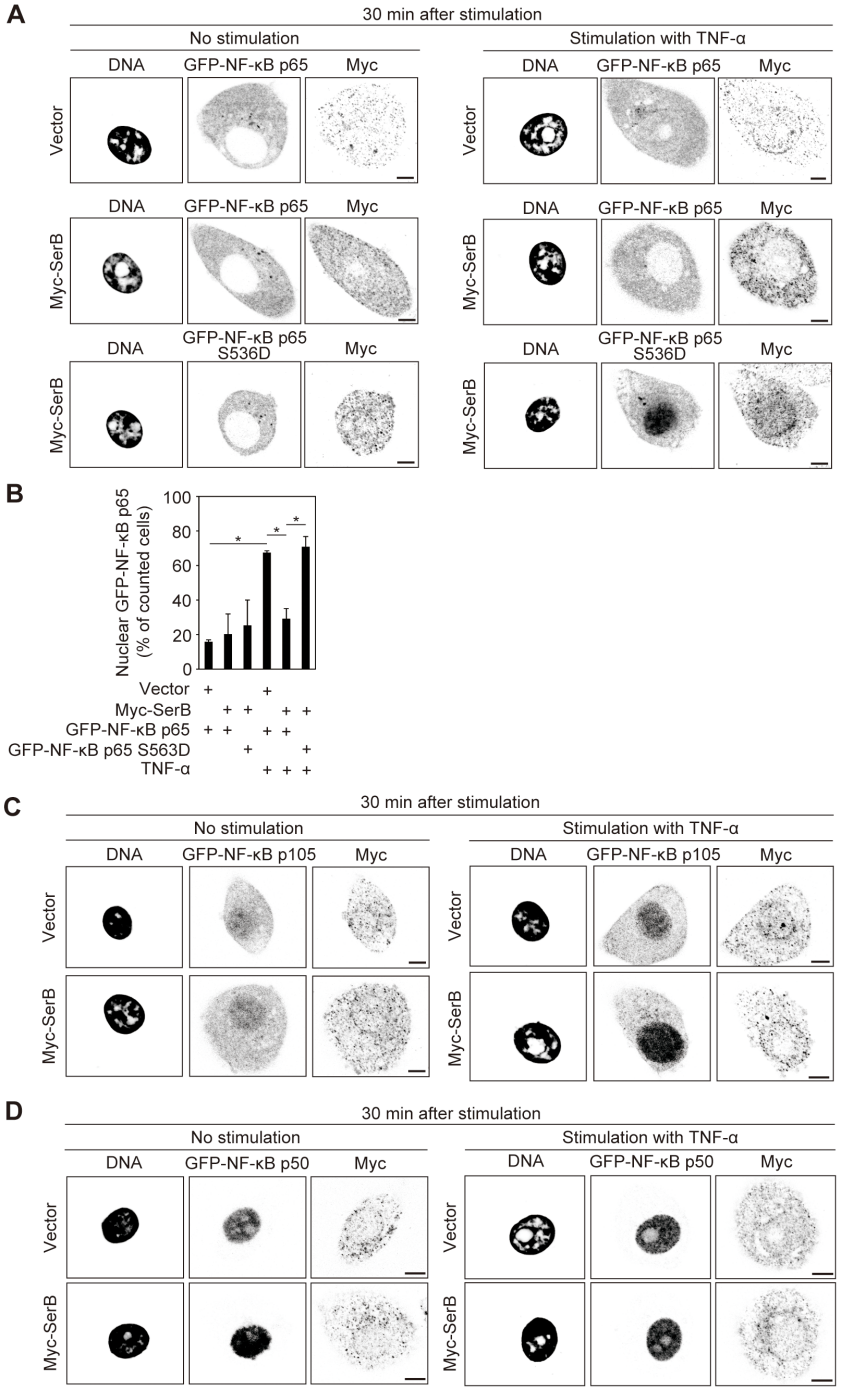
SerB inhibits nuclear translocation of NF-κB p65 but not p105/p50. (**A**) Confocal microscopy of TIGKs transiently co-transfected with Myc (Vector) or Myc-SerB along with GFP-NF-κB p65 or GFP-NF-κB p65 S536D and left unstimulated or stimulated with TNF-α (5 ng/ml) for 30 min. Cells were fixed and stained with DAPI and anti-Myc. Bars = 5 µm. Result is representative of 3 biological replicates. (**B**) Quantification of nuclear translocation in cells in A). Results are expressed as percentage of Myc positive cells with nuclear GFP- NF-κB p65 and are the mean with SEM of three independent experiments. At least 90 Myc/GFP positive cells were counted per test. *, p<0.05. (**C**) Confocal microscopy of TIGKs transiently co-transfected with Myc (Vector) or Myc-SerB along with GFP-NF-κB p105 and left unstimulated or stimulated with TNF-α (5 ng/ml) for 30 min. Cells were fixed and stained with DAPI and anti-Myc. Bars = 5 µm. Result is representative of 3 biological replicates. (**D**) Confocal microscopy of TIGKs transiently co-transfected with Myc (Vector) or Myc-SerB along with GFP-NF-κB p50 and left unstimulated or stimulated with TNF-α (5 ng/ml) for 30 min. Cells were fixed and stained with DAPI and anti-Myc. Bars = 5 µm. Result is representative of 3 biological replicates.

The phosphorylation status of NF-κB p65 Ser 536 has been shown to control the kinetics of NF-κB p65 nuclear import in Jurkat cells [20]. Based on this observation, we postulated that SerB can block the translocation of NF-κB p65 to the nucleus in stimulated cells. To test this idea, we transfected TIGKs with either GFP-tagged NF-κB p65 or GFP- tagged phosphomimic NF-κB p65 S536D. Cells were co-transfected with Myc-SerB and the location of GFP-NF-κB p65 determined by confocal microscopy. Upon stimulation with TNF-α, 68% of counted control cells were positive for GFP-NF-κB p65 in the nucleus, confirming nuclear translocation of the NF-κB p65 subunit (Figures 4A and 4B). However, in stimulated TIGKs expressing Myc-SerB and GFP-NF-κB p65 fusions, only 26% of counted cells were positive for GFP-NF-κB p65 in the nucleus. In cells expressing Myc-SerB and GFP-NF-κB p65 S536D, the number of counted cells positive for nuclear NF-κB p65 was similar to the level in cells without SerB, demonstrating that translocation of the constitutively active p65 subunit is unaffected by SerB. These results suggest that dephosphorylation of S536 by SerB inhibits nuclear translocation of NF-κB p65. As controls, we examined nuclear translocation of GFP-NF-κB p50 or GFP-NF-κB p105 fusions in TIGKs co-transfected with Myc-SerB. The presence of SerB did not affect the nuclear translocation of p50 or p105 (Figures 4C and 4D), further evidence that the action of SerB is specific for the p65 subunit.

### SerB inhibits the promoter response regulated by NF-κB p65

**Figure 5:**
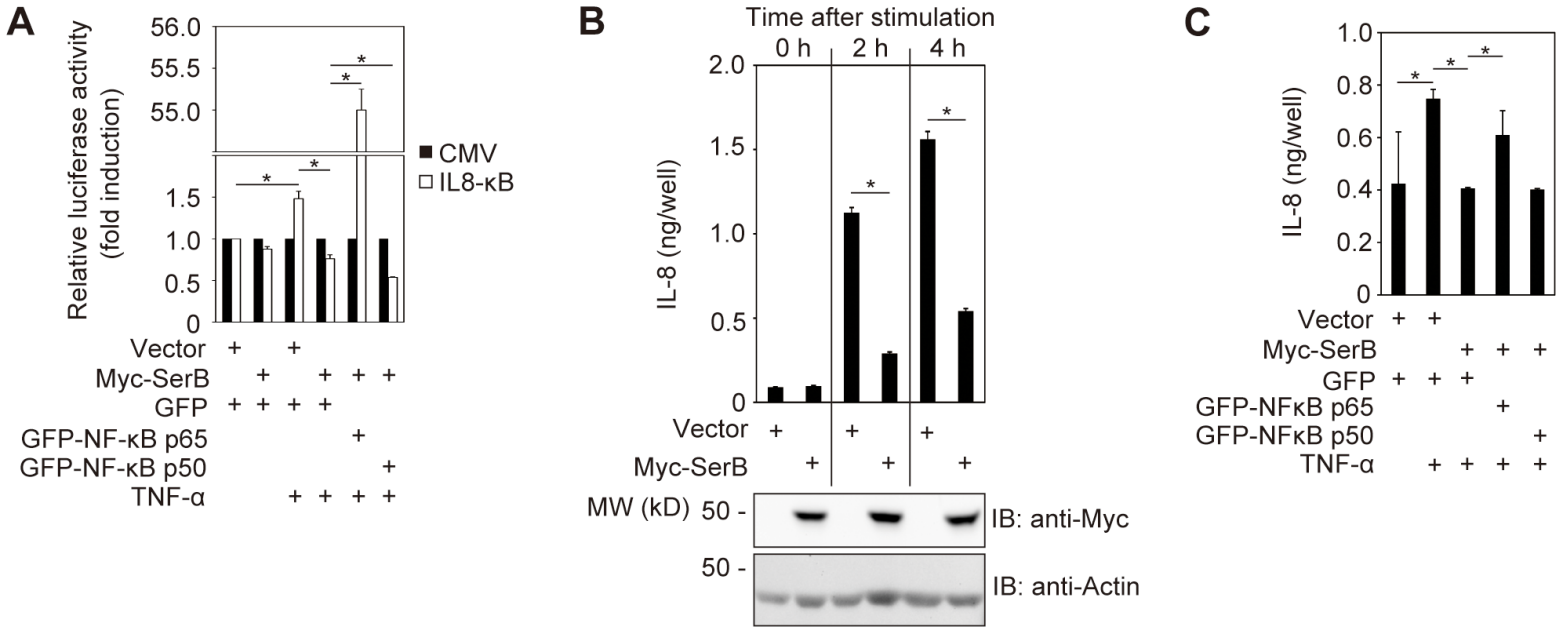
SerB dephosphorylation of p65 inhibits IL8 promoter activity and IL-8 production. (**A**) TIGKs were transiently co-transfected with Myc (Vector) or Myc-SerB, and either of GFP, GFP-NF-κB p65 or GFP-NF-κB p50, along with pIL-8 κB-Luc luciferase reporter for IL8 promoter activity or pRL-CMV Renilla luciferase control. Cells were stimulated with TNF-α (40 ng/ml) as indicated, and after 3 h TNF-α-induced IL8 κB luciferase activity was measured and normalized to Renilla luciferase. Results are presented as fold relative to the activity of the non-stimulated control and are means ± SD of 6 biological replicates. *, p<0.05. (**B**) TIGKs were transiently transfected with Myc (Vector) or Myc-SerB and at 36 h after transfection stimulated with TNF-α (5 ng/ml) as indicated. At the indicated time periods, the level of IL-8 in culture supernatants was measured by ELISA. Values are mean ± SD of 6 biological replicates. *, p<0.05. (**C**) TIGKs were transiently co-transfected Myc (Vector) or Myc-SerB, and either of GFP, GFP-NF-κB p65 or GFP-NF-κB p50. Cells were stimulated with TNF-α (5 ng/ml) as indicated, and after 4 h the level of IL-8 in culture supernatants was measured by ELISA. Values are mean ± SD of 6 biological replicates. *, p<0.05.

It is reported that phospho-NF-κB p65 does not associate with NF-κB p50, and that NF-κB p50-independent NF-κB p65 can be recruited to the IL8 promoter following stimulation by ionomycin in Jurkat cells [21]. We hypothesized, therefore, that dephosphorylation of NF-κB p65 by SerB would be sufficient to prevent transcription of the IL8 gene. To test this possibility, Myc-SerB and the luciferase IL8 reporter plasmid, pIL8κB-Luc, were co-expressed in TIGKs. In response to TNF-α, luciferase activity of cells expressing Myc-SerB was significantly reduced compared to cells transfected with empty vector (Figure 5A). We then examined the ability of excess exogenous p65 to partially relieve suppression of the IL8 reporter by introducing GFP-NF-κB subunits into the cells containing the reporter plasmid. Additional co-transfection of GFP-p65 with Myc-SerB increased promoter activity from 0.76- to 55-fold of the control level compared with cells expressing Myc-SerB and GFP at 3 h after stimulation by TNF-α (Figure 5A). In contrast, exogenous p50 subunit did not relieve SerB-mediated suppression of the IL8 promoter (Figure 5A) and indeed decreased luciferase activity indicating that overexpression of p50 subunits can interfere with the formation of p65 homodimers. Levels of TNF-α stimulated IL8 promoter activity in the presence of GFP-p65 and Myc-SerB were significantly lower than those obtained with GFP-p65 and Myc alone (Figure S6), consistent with the data in Figure 4.

To confirm the relevance of the interaction between SerB and NF-κB p65 for IL-8 production, IL-8 secreted into TIGK culture media was measured by ELISA. Two hours after TNF-α stimulation, IL-8 production was decreased by 74% in cells expressing Myc-SerB, and at 4 h after stimulation the IL-8 level was reduced by 65% (Figure 5B). Co-transfection with GFP-NF-κB p65 partially relieved the inhibition of TNF-α induced IL-8 secretion, whereas GFP-NF-κB p50 had no effect (Figure 5C).

### HAD superfamily motifs are required for the inhibitory activity of SerB on phosphorylation and nuclear translocation of NF-κB p65

**Figure 6:**
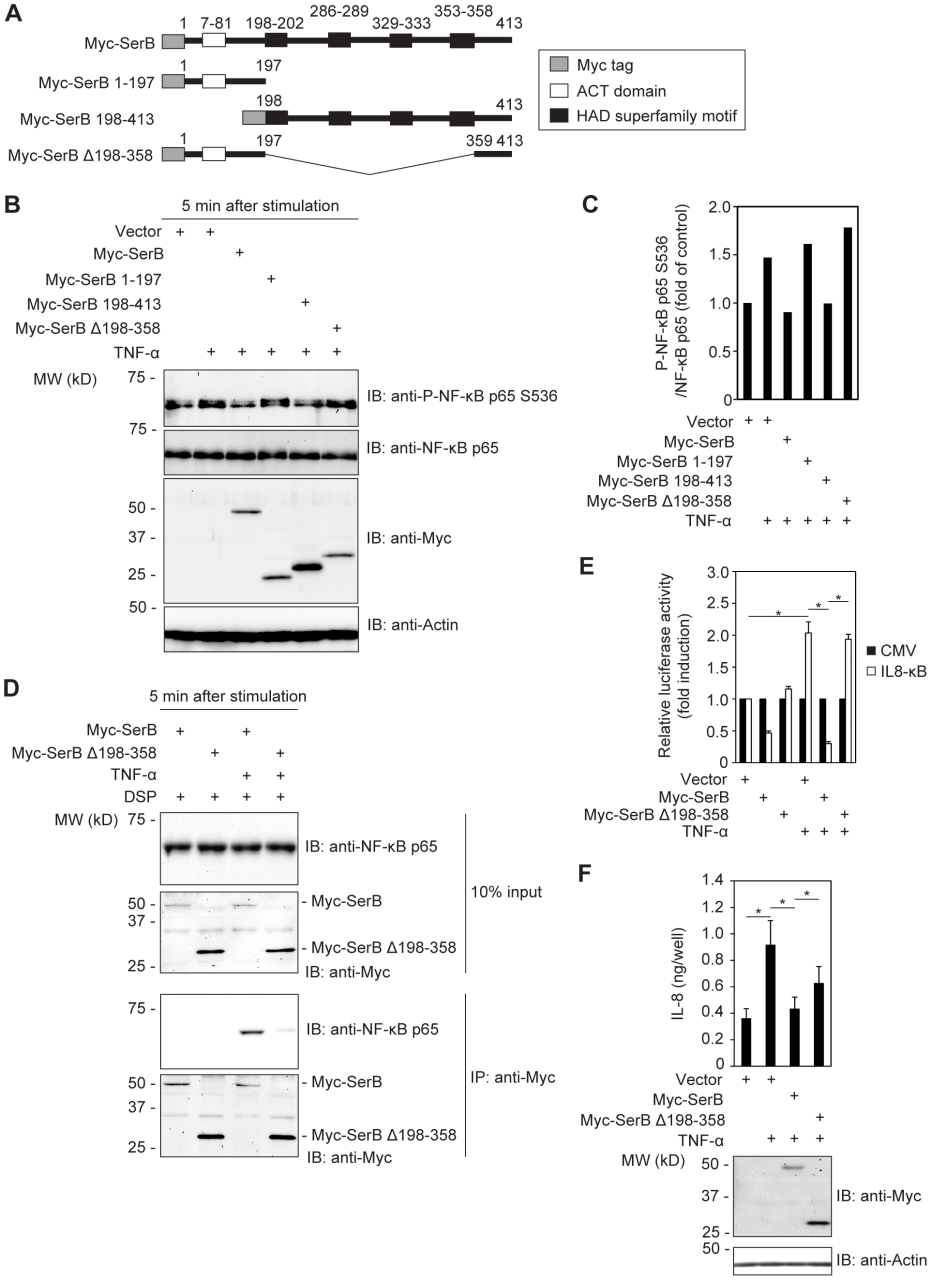
SerB HAD superfamily motifs are required for dephosphorylation of NF-κB p65. (**A**) Schematic view of the SerB structure and derivatives. ACT domains are indicated by white boxes and HAD superfamily motifs are shown with black boxes. (**B**) TIGKs transiently expressing SerB mutants were stimulated with TNF-α (5 ng/ml) for 5 min as indicated, and cell lysates immunoblotted (IB). Actin was used as a loading control. Result is representative of 2 biological replicates. (**C**) Densitometry of immunoblot in B) showing ratio of phospho-NF-κB p65 (S536) relative to total immunodetectable NF-κB p65. (**D**) TIGKs transiently expressing Myc-SerB or Myc-SerB Δ198–358 were stimulated with TNF-α (10 ng/ml) for 5 min or left unstimulated. Cells were cross-linked with DSP and cell lysates were immunoprecipitated with anti-Myc antibody. Immunoblots (IB) show cell lysates prior to immunoprecipitation (10% input) and immunoprecipitates (IP) with Myc antibodies. Result is representative of 3 biological replicates. (**E**) TIGKs were transiently co-transfected with Myc (Vector), Myc-SerB or Myc-SerB Δ198–358, and pIL-8 κB-Luc luciferase reporter plasmid for IL8 promoter activity or pRL-CMV control. Cells were stimulated with TNF-α (10 ng/ml) or left unstimulated, and after 3 h IL8 κB luciferase activity was measured and normalized to Renilla luciferase. Results are presented as fold relative to the activity of the non-stimulated control. Values are mean ± SD of 8 biological replicates. *, p<0.05. (**F**) TIGKs transiently expressing Myc (Vector), Myc-SerB or Myc-SerB Δ198-358 were stimulated with TNF-α (5 ng/ml) as indicated. At 4 h after stimulation, the level of IL-8 in culture supernatants was measured by ELISA (upper panel). Results are mean ± SD of 6 biological replicates. *, p<0.05. Cells were lysed and immunoblotted (IB) with Myc antibodies (lower panel) to confirm expression of Myc-SerB or Myc-SerB Δ198–358. Actin was used as a loading control.

SerB possesses 4 HAD family motifs which determine phosphatase activity [26], along with an ACT small molecule binding domain. To assess the contribution of these domains, we performed a structure-function analysis of SerB with deletion and truncation constructs lacking either the HAD domains or the ACT domain (Figure 6A). These constructs were expressed in TIGKs which were then stimulated with TNF-α to induce NF-κB p65 phosphorylation. As shown in Figures 6B and 6C, Myc-SerB 1–197 and Myc-SerB Δ198–358, which do not possess the HAD family motifs, were incapable of inducing dephosphorylation of NF-κB p65. In contrast, Myc-SerB 198–413 which lacks the ACT domain but not the HAD domains reduced the level of phospho-p65 to a similar level compared to full length SerB. Additionally, Myc-SerB Δ198–358 was unable to co-precipitate with NF-κB p65 (Figure 6D). Next we performed a luciferase reporter assay to examine the activity of Myc-SerB Δ198–358 on the IL8 promoter using reporter plasmid pIL8κB-Luc. Compared with cells expressing Myc-SerB, luciferase activity of cells expressing Myc-SerB Δ198-358 increased from 0.30- to 1.93-fold of the control following stimulation with TNF-α (Figure 6E). We also performed ELISA to quantify secreted IL-8 in supernatants of TIGKs expressing Myc-SerB Δ198-358. IL-8 secretion was significantly increased in cells expressing Myc-SerB Δ198-358 compared to cells expressing full length Myc-SerB (Figure 6F). These results illustrate the necessity of the HAD-family motifs for SerB phosphatase activity against NF-κB p65 S536, and for the consequent effects on transcription of the IL8 gene and the secretion of IL-8 induced by TNF-α.

**Figure 7:**
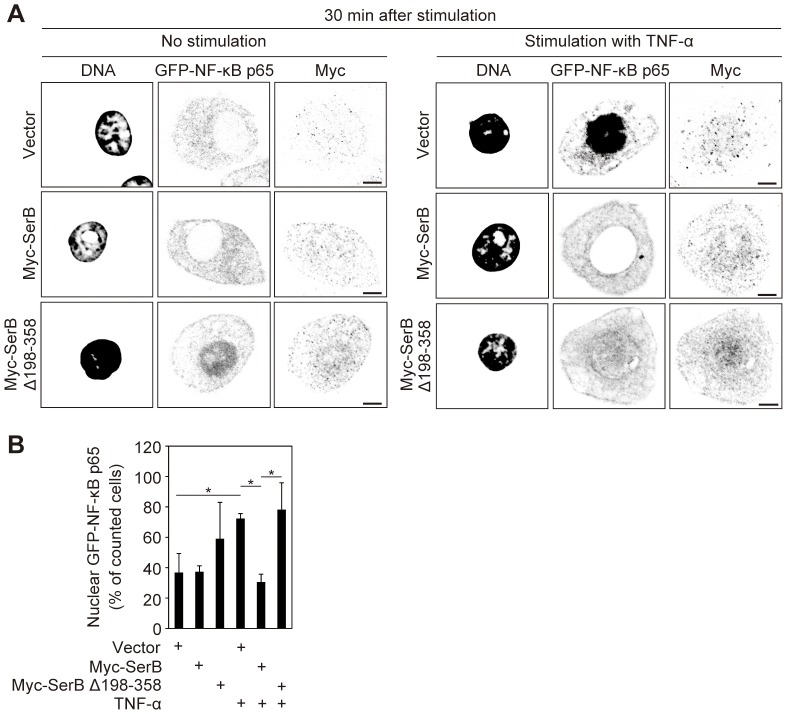
SerB HAD superfamily motifs are required for inhibition of nuclear translocation of NF-κB p65. (**A**) Confocal microscopy images of TIGKs transiently co-transfected with Myc (Vector), Myc-SerB or Myc-SerB Δ198-358 along with GFP-NF-κB p65 and left unstimulated or stimulated with TNF-α (5 ng/ml) for 30 min. Cells were fixed and stained with DAPI and anti-Myc. Bars = 5 µm. Result is representative of 3 biological replicates. (**B**) Quantification of nuclear translocation in cells transfected with Myc (Vector), Myc-SerB or Myc-SerB Δ198–358 along with GFP-NF-κB p65 and stimulated with TNF-α or left unstimulated. Results are expressed as percentage of Myc positive cells with nuclear GFP-NF-κB p65 and are the mean ± SD of 6 biological replicates. At least 90 Myc/GFP positive cells were counted per test. *, p<0.05.

To support the notion that the HAD-family motif is key for SerB activity, we performed immunofluorescence assays in TIGKs expressing GFP-NF-κB p65 and either of Myc-SerB or Myc-SerB Δ198–358. At 30 min after stimulation with TNF-α, 72% of counted cells transfected with empty vector were positive for GFP-NF-κB p65 in the nucleus (Figures 7A and 7B). In TIGKs expressing Myc-SerB, 30% of counted cells were positive for GFP-NF-κB p65 in the nucleus, while in cells expressing Myc-SerB Δ198–358, 78% of counted cells were positive, indicating that loss of the HAD-family motifs prevents SerB from blocking nuclear translocation of NF-κB p65.

Collectively these results indicate that SerB binds to the NF-κB p65 subunit in TIGKs and through the action of the HAD-family motifs dephosphorylates the S536 residue. Dephosphorylation of NF-κB p65 impedes nuclear translocation and activity of NF-κB p65 on the IL8 promoter, which is the mechanism that causes abrogation of IL-8 production by *P. gingivalis*.

## Discussion

Periodontal diseases are characterized by destruction of the gingival tissues including the alveolar bone, with eventual exfoliation of the tooth. These are among the most common infections of humans, and in developed countries over half of the adult population will experience some form of periodontitis [27]. The disease initiates at the epithelial surfaces of the subgingival compartment, and gingival epithelial cells play a central role in responding to microbial infection and orchestrating immune responses [1]. Mucosal inflammatory responses involve coordinated expression of cytokines and chemokines, and the NF-κB transcriptional regulator exerts control over a number of inflammation-associated genes.

*P. gingivalis* is a major pathogen in the initiation and progression of severe and chronic forms of periodontal disease. A feature of *P. gingivalis* infection is dysregulation of innate immunity [3], including suppression of IL-8 production from gingival epithelial cells. The functionally versatile serine phosphatase SerB of *P. gingivalis* is required for optimal invasion of *P. gingivalis* into epithelial cells [6], [8] and is also necessary for IL-8 suppression [9]. In this report we provide several lines of evidence that SerB antagonizes IL-8 production through dephosphorylation of the serine 536 residue of the RelA/p65 subunit of NF-κB. This evidence includes: infection of epithelial cells with a *P. gingivalis* Δ*serB* mutant induces less dephosphorylation of NF-κB p65 S536 and higher levels of IL8 promoter activity compared to parental *P. gingivalis*; ectopically expressed SerB binds to and dephosphorylates p65 in epithelial cells stimulated with TNF-α; exogenous SerB impedes nuclear translocation of p65; and SerB blocks IL8 promoter activity and secretion of IL-8 from epithelial cells.

Given the role of NF-κB in directing cytokine production and innate immune responses, it is not surprising that many human pathogens have evolved sophisticated mechanisms to interfere with NF-κB activity and disrupt host immunity [28]. Bacterial disruption of NF-κB can occur at multiple points in the activation pathway. Several organisms target the IKK complex, a central node that integrates the input from a number of upstream pathways. For example, the InlC internalin of *Listeria monocytogenes* can bind to IKKα and block IκBα phosphorylation [29], while the NleH1 and NleH2 proteins of EHEC impede IκB ubiquitination [30]. Specific bacterial effectors can also target TLR signaling upstream of the IKK complex [31], and NF-κB-dependent transcription downstream of IKK [32]. While the latter mechanism is less commonly recognized, it is less likely to impact other signaling pathways that intersect with TLR signaling and with the IKK complex [33], and thus provide a more precise redirection of host immune responses. For example, the NleH1 effector of EHEC also binds to NF-κB RPS3 and prevents phosphorylation [32]. NleH1 can thus selectively reduce the transcription of RPS3-dependent target genes, including IL8 and TNF, without attenuating promoter activity of other NF-κB-dependent genes. The ability of *P. gingivalis* SerB to prevent nuclear translocation of p65 is a previously unrecognized bacterial NF-κB subversion strategy involving specific dephosphorylation of a NF-κB subunit.

Phosphorylation of p65 at S536 can elevate nuclear translocation and transcriptional activity of NF-κB [34]. However, the full extent of NF-κB regulation that is dependent on serine phosphorylation of p65 remains to be fully defined, and may vary according to cell type and phosphorylation mechanism [17]. Various studies have shown both inducible phosphorylation of S536 and a pool of constitutively phosphorylated p65 [21], [35]. Our data show that in gingival epithelial cells, S536 phosphorylation is induced by TNF-α and that nuclear translocation of p65 is enhanced by phosphorylation. Immunoblot analyses did not reveal a pool of phospho-p65 in the absence of stimulation, similar to the situation in intestinal epithelial cells [36].

Among the pathogenic constituents of the periodontal polymicrobial community, *P. gingivalis* has a unique ability to selectively antagonize IL-8 production in epithelial cells, even in the context of co-infection with other stimulatory bacteria [2], [9]. Conversely, *P. gingivalis* can induce the secretion of other proinflammatory cytokines [37]. These properties are consistent with a specific action of *P. gingivalis* on NF-κB p65. It has been reported that the IL8 promoter is regulated by p65 homodimers, and that neither p105/p50 homodimers nor p105/p50 - p65 heterodimers, can activate transcription from the IL8 promoter [38]. In addition, in this study we found that overexpression of NF-κB p65, but not p50, induces the secretion of IL-8 (Figure 5C). Crystallization studies show that NF-κB p65 homodimers bind to a pseudosymmetric sequence in the enhancer region of the IL8 gene [39]. Moreover, phospho-p65 can be selectively recruited to the NF-κB site on the IL8 promoter independent of p50 [21]. Hence, dephosphorylation of p65 and inhibition of nuclear translocation will allow *P. gingivalis* to selectively manipulate different aspects of innate immunity. NF-κB also controls expression of a number of host genes that govern cell proliferation and programmed cell death [10], and thus broadly based inhibition of NF-κB would be predicted to elevate apoptotic cell death. *P. gingivalis*, however, suppresses apoptotic cell death in epithelial cells and accelerates progression through the cell cycle [40]. Collectively these results point toward an exquisite specificity of the interaction between *P. gingivalis* and NF-κB, allowing process-specific manipulation.

*P. gingivalis* is an intracellular organism which lacks the machinery and effector molecules of the type III secretion system that are utilized by many invasive pathogens [41]. Rather, *P. gingivalis* relies on a more limited number of multifunctional effectors, such as the HAD family SerB, to direct entry into host epithelial cells [8]. While the SerB deficient mutant of *P. gingivalis* has a diminished capacity to invade and survive within epithelial cells [8], the ability of ectopically expressed SerB to dephosphorylate p65 establishes that abrogated p65 dephosphorylation by the SerB mutant is not a reflection of lower levels of intracellular bacteria, but due to the absence of the enzyme-substrate reaction. Furthermore, although SerB can dephosphorylate a variety of host cell proteins, such as cofilin [6], its impact on the NF-κB pathway appears to be restricted to the p65 subunit. The effect of SerB on p65 phosphorylation, nuclear translocation and transcription of the IL8 gene all require the HAD enzyme domains. Eukaryotic HAD family enzymes have been shown to regulate innate immunity by modulating the phosphorylation state of signal transducers involved in host responses to viral infection [42]; however, this is the first example of a bacterial HAD family phosphatase that can divert innate immunity through dephosphorylation of host signaling molecules. The ability of *P. gingivalis* SerB to specifically target IL8 transcription through dephosphorylation of p65 provides a molecular basis to the localized chemokine paralysis induced by *P. gingivalis* at mucosal surfaces. Many bacteria possess SerB homologs which are generally annotated as metabolic enzymes. It will be interesting to discover whether secretion of this enzyme and activity against NF-κB are features of pathogens, particularly keystone pathogens in mixed communities.

## Materials and Methods

### Bacterial strains, eukaryotic cells and growth conditions

Wild-type (WT) *P. gingivalis* ATCC 33277, isogenic Δ*serB* and *serB*::FLAG were cultured anaerobically as described previously [6], [8]. TIGKs, telomerase immortalized gingival epithelial cells were generated by immortalization of primary gingival epithelial cells [22] with bmi1/hTERT [43], and were maintained in Keratinocyte-SFM (Invitrogen) supplemented with 5 ng/ml Human Recombinant Epidermal Growth Factor and 50 µg/ml Bovine Pituitary Extract.

### Antibodies and reagents

Mouse monoclonal anti-Myc and mouse monoclonal anti-β-actin were from Sigma-Aldrich; mouse monoclonal anti-NF-κB p65, rabbit monoclonal anti-phospho-NF-κB p65 S536, rabbit monoclonal anti-NF-κB p65 S468, rabbit monoclonal anti-NF-κB p105/p50, rabbit monoclonal anti-phospho-NF-κB p105 S933, and rabbit monoclonal anti-calnexin were from Cell Signaling Technology; rabbit polyclonal anti-NF-κB p65 S276 was from Abcam; rabbit monoclonal anti-β-tubulin was from Imgenex; and mouse monoclonal anti-FLAG was from Invitrogen. *P. gingivalis* whole cell antibody and SerB antibodies have been described previously [6], [9]. Rhodamine Red-X-conjugated secondary antibody (goat anti-mouse IgG) and Alexa Fluor 488-conjugated secondary antibody (goat anti-rabbit IgG) from Invitrogen were used for fluorescent microscopy. Horseradish peroxidase (HRP)-conjugated secondary antibodies (goat anti-mouse IgG and goat anti-rabbit IgG, Cell Signaling Technology) were used for immunoblotting. FITC-conjugated phalloidin (Sigma-Aldrich) was used to stain actin for fluorescent microscopy. TNF-α (PeproTech) was used for cell stimulation.

### Transient transfection

A chimeric construct of Myc-SerB was constructed by cloning PCR amplified *serB* from *P. gingivalis* into pCMV-Myc (Clontech) using exogenously added *EcoRI* sites. Myc-tagged SerB mutants consisting of amino acids 1–197, 198–413 and a deletion of amino acids 198–358 were produced using PCR and inserted into pCMV-Myc using exogenously added *EcoRI* sites. GFP-tagged NF-κB p65 was produced from TIGK cDNA and inserted into pAcGFP1-C1 (Clontech) using exogenously added *KpnI* sites. A point mutation of GFP-tagged NF-κB p65 to S536D was introduced by overlapping fusion PCR. GFP-tagged NF-κB p105 was produced from TIGK cDNA and inserted into pAcGFP1-C1 using exogenously added *EcoRI* sites. GFP-tagged NF-κB p50 was produced from amino acids 1 to 438 of NF-κB p105 and cloned into pAcGFP1-C1 according to the published sequence [44]. The pIL-8 κB-Luc reporter plasmid was designed according to the published sequence [38]. The enhancer sequence of IL8 was cloned into pGL4.23 [luc2/minP] (Promega) using exogenously added *KpnI* and *NheI* sites. Primer sequences are listed in Table S1. All PCR products and mutations were confirmed by sequencing. Transient transfection of TIGK cells was performed using FuGENE 6 Transfection Reagent (Promega).

### Confocal microscopy

TIGKs were fixed with 3% paraformaldehyde in PBS for 30 min at room temperature, permeabilized with 0.1% Triton X-100 in PBS for 5 min at room temperature and blocked with 0.1% gelatin in PBS for 20 min at room temperature. Primary antibodies were diluted 1∶400 in PBS, and Rhodamine Red-X-conjugated secondary antibodies were diluted 1∶400 in PBS. Antibody incubations were for 1 h at room temperature, followed by six washes in PBS. Cells were mounted onto glass slides using Vectashield Mounting Medium with DAPI (Vector laboratories) to label the bacterial and cellular DNA, and examined using laser scanning confocal microscopy (FV1000; Olympus). Images acquired and analyzed using FluoView software (Olympus).

### NF-κB nuclear translocation

TIGKs were transiently transfected for 36 h and stimulated with TNF-α for 30 min (except where indicated). GFP and Myc tags were detected by confocal microscopy. At least 90 GFP/Myc positive cells were counted in each condition.

### Isolation of membrane and cytosolic fractions

TIGKs were washed with PBS and suspended in homogenization buffer (3 mM imidazole [pH 7.4], 250 mM sucrose, 0.5 mM EDTA). Cells were mechanically disrupted by vigorous passage through 23- and 27-gauge needles 8 times on ice. The sample was centrifuged at 3,000 *g* for 15 min at 4°C to remove the pellet of bacteria, unbroken TIGKs, host nuclei and cytoskeletal components. The sample was further centrifuged at 17,400 *g* for 30 min at 4°C and the supernatant was used as the cytosolic fraction. The pellet was lysed in lysis buffer (2 M thiourea, 7 M urea, 3% CHAPS, 1% Triton X-100) on ice for 30 min. After centrifugation at 17,400 *g* for 30 min at 4°C, the supernatant was used as the membrane fraction.

### Immunoblotting

Cells were lysed, clarified by centrifugation, separated by SDS-PAGE and transferred to nitrocellulose membranes. Membranes were blocked with PBST (PBS and 0.1% Tween 20) containing 1% skim milk for 1 h at room temperature, and were then incubated for 1 h at room temperature with primary antibodies diluted in PBST. Membranes were washed three times with PBST, incubated for 1 h at room temperature with a 1∶5,000 dilution of HRP-conjugated secondary antibodies in PBST, and washed three times with PBST. Immunoreactive bands were detected using Pierce ECL Western Blotting Substrate (Thermo Scientific) and ChemiDoc XRS Plus (Bio-Rad). Images were acquired with Image Lab Software version 3.0 (Bio-Rad).

### Cross-linking

Cells were cross-linked with 0.25 mM dithiobis (DSP, Thermo Scientific) in PBS for 5 min at room temperature. After washing cells with quenching buffer (1 M glycine in PBS [pH 7.4]) 4 times, cell proteins were extracted in lysis buffer.

### Immunoprecipitation

Cell lysates were subjected to pull down reactions using the Anti-c-Myc Immunoprecipitation Kit (Sigma-Aldrich) according to the manufacturer's protocol. Proteins bound to the anti-c-Myc agarose were analyzed by immunoblotting.

### Luciferase reporter assay

TIGKs were transfected with pIL-8 κB-Luc, pRL-CMV Vector (Promega), and various combinations of expression plasmids. Total plasmid amounts were equalized in each transfection. Cells were lysed and reporter activity was determined using the Dual-Glo Luciferase Assay System (Promega). Firefly luciferase activity was normalized on the basis of Renilla luciferase activity in the same extracts.

### ELISA

IL-8 concentrations in TIGK culture supernatants were determined using the Quantikine Human CXCL/IL-8 (R&D Systems) according to manufacturer's protocol.

### Statistical analyses

p-value was determined using two-tailed *t* test (closed testing procedure) and p<0.05 was considered significant.

## Supporting Information

Figure S1 Comparison of antibody and DAPI staining of intracellular *P. gingivalis*. Confocal microscopy images of TIGKs infected with *P. gingivalis* expressing FLAG-SerB at a MOI of 10. At 2 h after infection, cells were fixed and stained with DAPI (cyan) or *P. gingivalis* antibodies (green). Bars = 5 µm. Result is representative of 2 biological replicates.
(PDF)

Figure S2 SerB can be found in the cytosol and in the membrane of *P. gingivalis* infected cells. TIGKs were infected with *P. gingivalis* WT or Δ*serB* at MOI 50. After 3 h, cytoplasmic and membrane fractions were prepared and immunoblotted with the antibodies indicated. Calnexin was used as a membrane marker, and β-tubulin was used as a cytosol marker. Result is representative of 2 biological replicates.
(PDF)

Figure S3 Ectopically expressed SerB binds to NF-κB p65. TIGKs transiently expressing Myc or Myc-SerB were immunoprecipitated with anti-Myc antibody. Left panel is immunoblot (IB) of cell lysate prior to immunoprecipitation. Right panel is blot of immunoprecipitate (IP). Result is representative of 2 biological replicates.
(PDF)

Figure S4 Effects of ectopic expression of SerB on the phosphorylation of NF-κB p65 S276. (A) TIGKs were transfected with empty vector or Myc-SerB. After 36 h, cells were stimulated with TNF-α (5 ng/ml) and at the indicated time periods cell extracts were prepared and immunoblotted with the antibodies shown. Actin was used as a loading control. Result is representative of 2 biological replicates. (B) Densitometry of immunoblot in A) showing ratio of phospho-NF-κB p65 (S276) relative to total immunodetectable NF-κB p65.
(PDF)

Figure S5 Effects of ectopic expression of SerB on the phosphorylation of NF-κB p65 S468. (A) TIGKs were transfected with empty vector or Myc-SerB. After 36 h, cells were stimulated with TNF-α (5 ng/ml) and at the indicated time periods cell extracts were prepared and immunoblotted with the antibodies shown. Actin was used as a loading control. Result is representative of 2 biological replicates. (B) Densitometry of immunoblot in A) showing ratio of phospho-NF-κB p65 (S468) relative to total immunodetectable NF-κB p65.
(PDF)

Figure S6 Effects of overexpression of SerB and NF-κB p65 on IL8 promoter activity. TIGKs were transiently co-transfected with Myc (Vector) or Myc-SerB, and pIL-8 κB-Luc or pRL-null as an internal control. Cells were stimulated with TNF-α (10 ng/ml) as indicated, and after 3 h TNF-α-induced IL8 κB promoted luciferase activity was measured. Results are presented as fold induction relative to the activity of the non-stimulated control and are means ± SD of 6 biological replicates. *, p<0.05.
(PDF)

Table S1 Primers used in this study.
(PDF)
