# Supplementary figures and images for: The Serine Phosphatase SerB of Porphyromonas gingivalis Suppresses IL-8 Production by Dephosphorylation of NF-κB RelA/p65
Source: PLoS Pathog. 2013 Apr 18;9(4):e1003326. doi: 10.1371/journal.ppat.1003326 (PMC3630210; doi:10.1371/journal.ppat.1003326)

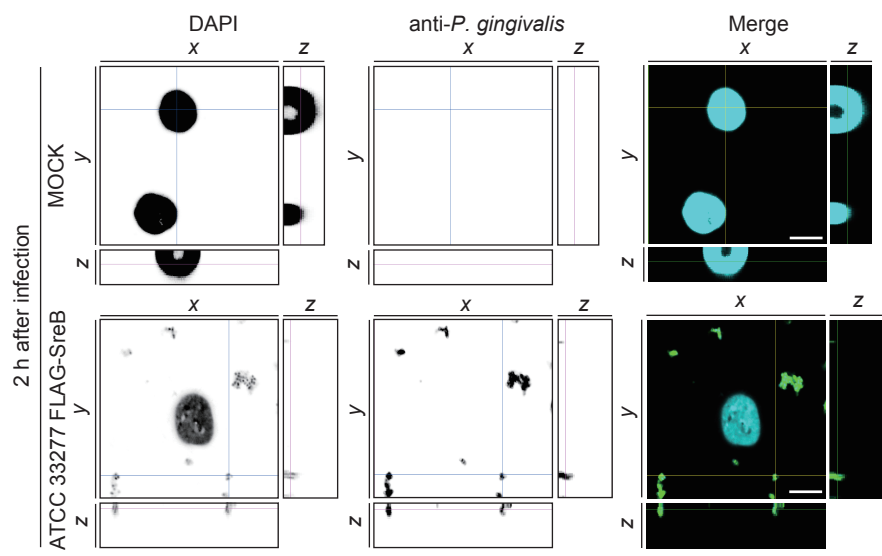

Supplement: Figure S1 — Comparison of antibody and DAPI staining of intracellular P. gingivalis. Confocal microscopy images of TIGKs infected with P. gingivalis expressing FLAG-SerB at a MOI of 10. At 2 h after infection, cells were fixed and stained with DAPI (cyan) or P. gingivalis antibodies (green). Bars = 5 µm. Result is representative of 2 biological replicates. (PDF) [file ppat.1003326.s001.pdf]

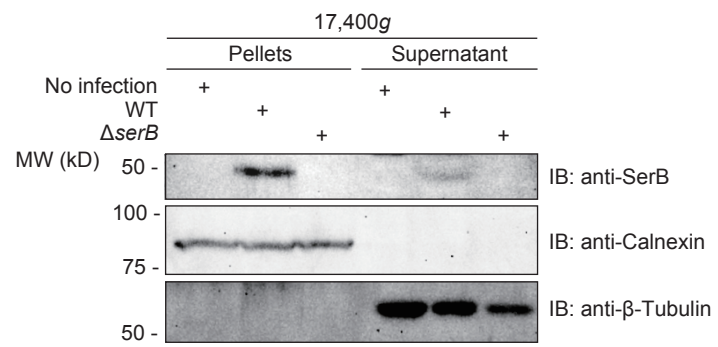

Supplement: Figure S2 — SerB can be found in the cytosol and in the membrane of P. gingivalis infected cells. TIGKs were infected with P. gingivalis WT or ΔserB at MOI 50. After 3 h, cytoplasmic and membrane fractions were prepared and immunoblotted with the antibodies indicated. Calnexin was used as a membrane marker, and β-tubulin was used as a cytosol marker. Result is representative of 2 biological replicates. (PDF) [file ppat.1003326.s002.pdf]

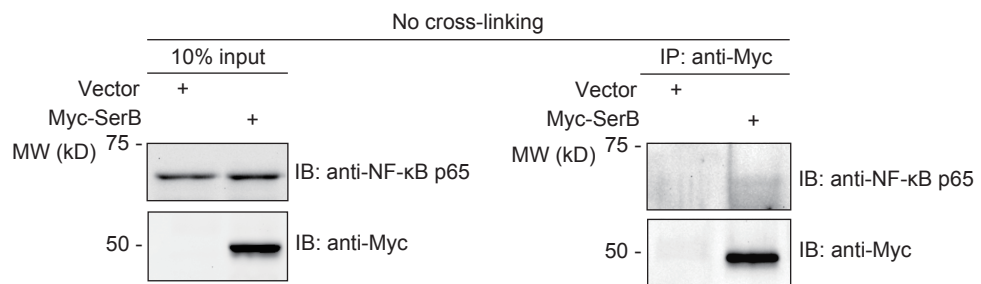

Supplement: Figure S3 — Ectopically expressed SerB binds to NF-κB p65. TIGKs transiently expressing Myc or Myc-SerB were immunoprecipitated with anti-Myc antibody. Left panel is immunoblot (IB) of cell lysate prior to immunoprecipitation. Right panel is blot of immunoprecipitate (IP). Result is representative of 2 biological replicates. (PDF) [file ppat.1003326.s003.pdf]

**A**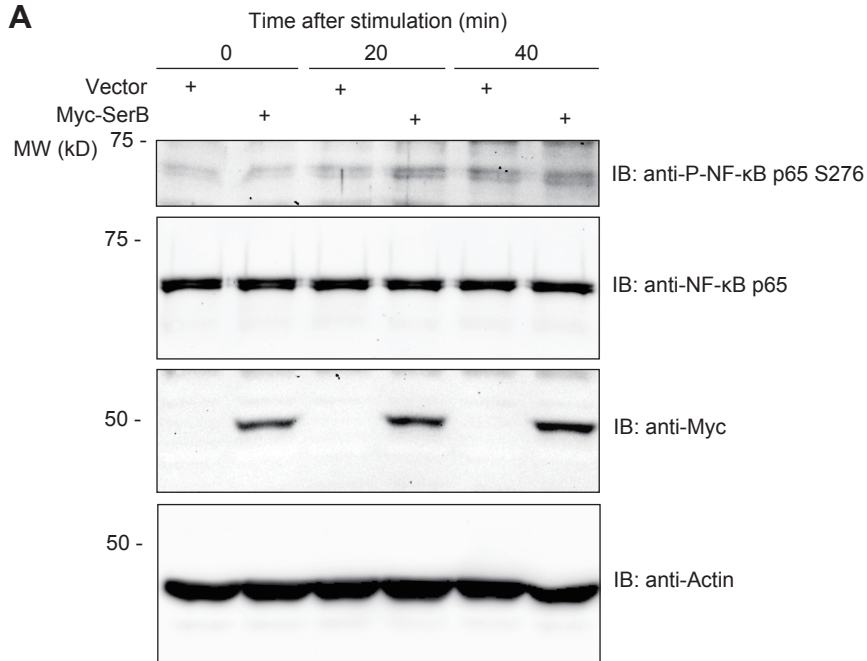**B**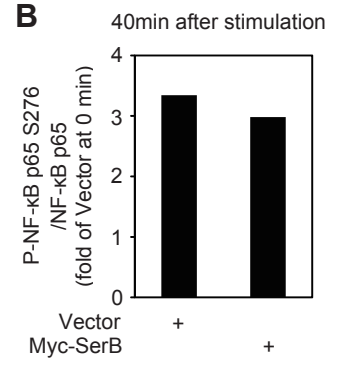

Supplement: Figure S4 — Effects of ectopic expression of SerB on the phosphorylation of NF-κB p65 S276. (A) TIGKs were transfected with empty vector or Myc-SerB. After 36 h, cells were stimulated with TNF-α (5 ng/ml) and at the indicated time periods cell extracts were prepared and immunoblotted with the antibodies shown. Actin was used as a loading control. Result is representative of 2 biological replicates. (B) Densitometry of immunoblot in A) showing ratio of phospho-NF-κB p65 (S276) relative to total immunodetectable NF-κB p65. (PDF) [file ppat.1003326.s004.pdf]

**A**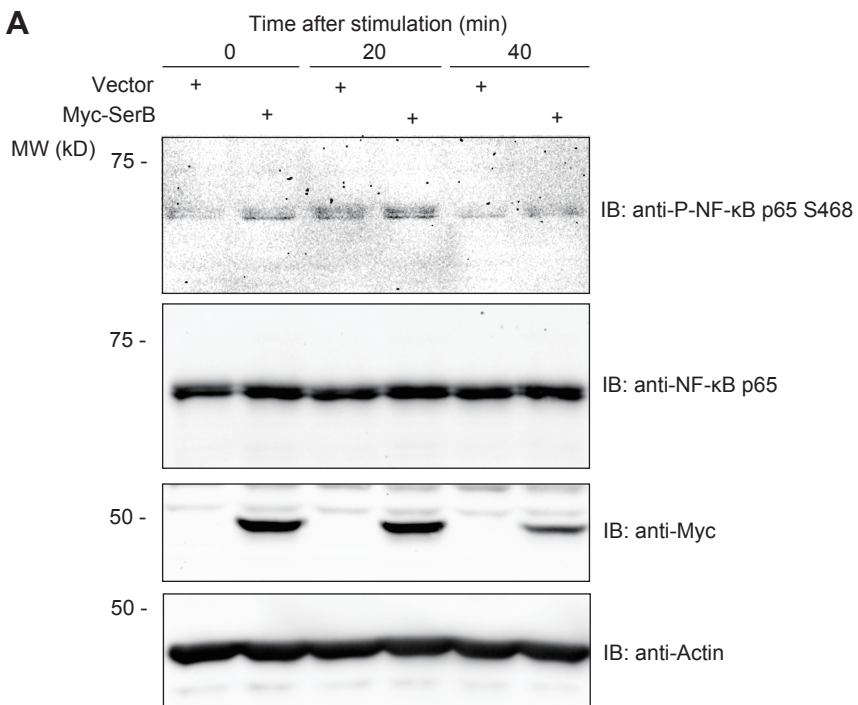**B**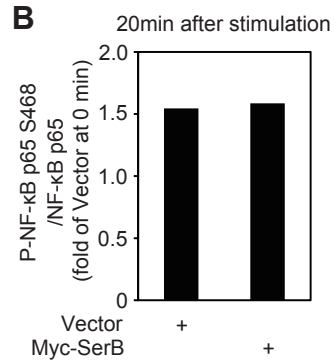

Supplement: Figure S5 — Effects of ectopic expression of SerB on the phosphorylation of NF-κB p65 S468. (A) TIGKs were transfected with empty vector or Myc-SerB. After 36 h, cells were stimulated with TNF-α (5 ng/ml) and at the indicated time periods cell extracts were prepared and immunoblotted with the antibodies shown. Actin was used as a loading control. Result is representative of 2 biological replicates. (B) Densitometry of immunoblot in A) showing ratio of phospho-NF-κB p65 (S468) relative to total immunodetectable NF-κB p65. (PDF) [file ppat.1003326.s005.pdf]

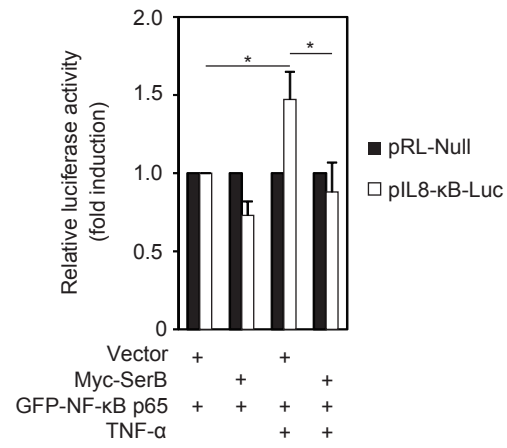

Supplement: Figure S6 — Effects of overexpression of SerB and NF-κB p65 on IL8 promoter activity. TIGKs were transiently co-transfected with Myc (Vector) or Myc-SerB, and pIL-8 κB-Luc or pRL-null as an internal control. Cells were stimulated with TNF-α (10 ng/ml) as indicated, and after 3 h TNF-α-induced IL8 κB promoted luciferase activity was measured. Results are presented as fold induction relative to the activity of the non-stimulated control and are means ± SD of 6 biological replicates. *, p<0.05. (PDF) [file ppat.1003326.s006.pdf]
