# Supplementary material for: The Serine Phosphatase SerB of Porphyromonas gingivalis Suppresses IL-8 Production by Dephosphorylation of NF-κB RelA/p65
Source: PLoS Pathog. 2013 Apr 18;9(4):e1003326. doi: 10.1371/journal.ppat.1003326 (PMC3630210; doi:10.1371/journal.ppat.1003326)
Supplement: Table S1 — Primers used in this study. (PDF) [file ppat.1003326.s007.pdf]

Name	Sequence
SerB-F	5'-CCGGAATTCTTATGAGTACAACACGA-3'
SerB-R	5’-CCGGAATTCCTATAATTTTTCCGTAAGCA-3’
SerB 1-197-R	5’-CCGGAATTCTTAGAAGCAAATGAGAC-3’
SerB 198-413-F	5’-CCGGAATTCTTGATATGGACTCTACCC-3’
SerB Δ198-358-F	5’-TCATTTGCTTCCTACCTATGATTGG-3’
SerB Δ198-358-R	5’-CCAATCATAGGTAGGAAGCAAATGA-3’
NF-κB p65-F	5’-CGGGGTACCATGGACGAACTGTTC-3’
NF-κB p65-R	5’-CGGGGTACCTTAGGAGCTGATCTGACTC-3’
NF-κB p65 S536D-F	5’-AGACTTCTCCGACATTGCGGACAT-3'
NF-κB p65 S536D-R	5’-ATGTCCGCAATGTCGGAGAAGTCT-3’
NF-κB p105-F	5’-CCGGAATTCTTATGGCAGAAGATGATCC-3’
NF-κB p105-R	5’-CCGGAATTCCTAAATTTTGCCTTCTAGAGG-3’
NF-κB p50-R	5’-CCGGAATTCAGTGTCCATGGTTCC-3’
IL-8 κB-F	5’-CGGGGTACC(TGTGGAATTTCCGC)$_4$GCTAGCTAG-3’
IL-8 κB-R	5’-CTAGCTAGC(GCGGAAATTCCACA)$_4$GGTACCCCG-3’
